# Effects of a web-based follow-up intervention on self-efficacy in obesity treatment for women

**DOI:** 10.1007/s00508-017-1198-7

**Published:** 2017-04-13

**Authors:** Sonja Rader, Thomas Ernst Dorner, Rudolf Schoberberger, Hilde Wolf

**Affiliations:** 1 Women’s Health Centre – FEM Süd, Kaiser Franz Josef – Hospital Vienna, Kundratstrasse 3, 1100 Vienna, Austria; 2 Institute of Social Medicine, Centre for Public Health, Medical University Vienna, Vienna, Austria

**Keywords:** Obesity, Self-efficacy, Web-based intervention, Follow-up support, e-Health

## Abstract

Obesity is a chronic disease requiring long-term care. The purpose of the current study was the evaluation of a web-based intervention (WBI), subsequent to an initial face to face life style treatment. In a randomized trial, 84 women received an introduction phase (4 months) and a training phase (2 months) where one group was trained in using WBI whereas the other arm received a printed manual (PMI). During the self-monitoring phase (6 months) participants either used the WBI or the PMI for follow-up support. Anthropometric parameters could be significantly reduced and self-efficacy was significantly increased in the first 6 months. At 12 months, values of self-efficacy of the WBI were not superior compared to results of the PMI; however, feedback on acceptability of the intervention did show higher ratings of the WBI and also facilitated contact with the program supervisor. No significant differences regarding the engagement in follow-up tools could be found between the intervention groups. Subgroup analysis indicated a positive effect of involvement in both forms of self-monitoring aftercare.

## Introduction

According to the World Health Organization (WHO), worldwide the number of overweight or obese adults (aged 18 years and older) exceeded 1.9 bn (1.9 ✕ 10^9^) in 2014 and 600 million (600 ✕ 10^6^) of this population were classified as obese [1]. Due to the complex interactions between genetic and epigenetic, behavioral, social and environmental factors, experts recommend long-term support after a weight reduction to reduce relapse [2–5]. It has been established that interventions, including self-efficacy training, adaption of outcome expectations and self-regulatory behavior, provide individuals with valuable requirements to maintain the desired long-term behavioral changes [2, 6]. It has to be understood that it is not enough to merely decide to start a new behavior. People with failed attempts at weight reduction need to be supported to regain the perception of control [7, 8]. Self-efficacy is targeted in general health promotion as well as in weight loss and stabilization. Individuals with a higher self-efficacy are more adherent to their decisions and although they choose more challenging tasks, their goal setting strategies are better balanced. Even in adverse conditions high self-efficacy is a protective factor to handle setbacks with greater success. If self-efficacy is specifically trained, coping strategies can even be more accurate and adapted to situations [8–10].

The gradual reduction of controlled treatment and the increase of autonomy are important in long-term follow-up support [11]. Studies of Statistics Austria call attention to new technological means with the potential to support tailored life style interventions. A high acceptance of mobile phone apps is documented as well as the increase of internet access via smartphones and tablets [12]. Especially healthcare strategies in treatment of chronic conditions have to incorporate empowering and self-help approaches, which profit from convenient self-monitoring tools, such as automatic reminders for enhanced skill training. Social media and blogs are tested in peer to peer communication or maintenance of regular contact with a professional healthcare provider [11, 13, 14]. However promising some of these new technical solutions might be, treatment settings have to consider existing gaps in internet access, availability of mobile devices, individual preferences and concerns. Hence electronic health strategies are best considered enhancements of established treatment plans or as add-on concepts [15, 16].

With the design of the study, the feasibility of a combination of a conventional face to face life style intervention and a web-based aftercare support was evaluated. Presented results are part of a larger study conducted as a doctoral thesis at the Medical University of Vienna, also assessing sustainability parameters and attrition including a historical control group. For comprehensibility, this article focuses on the primary endpoints, changes in nutrition and exercise self-efficacy.

## Material and methods

### Participants and recruitment

**Fig. 1:**
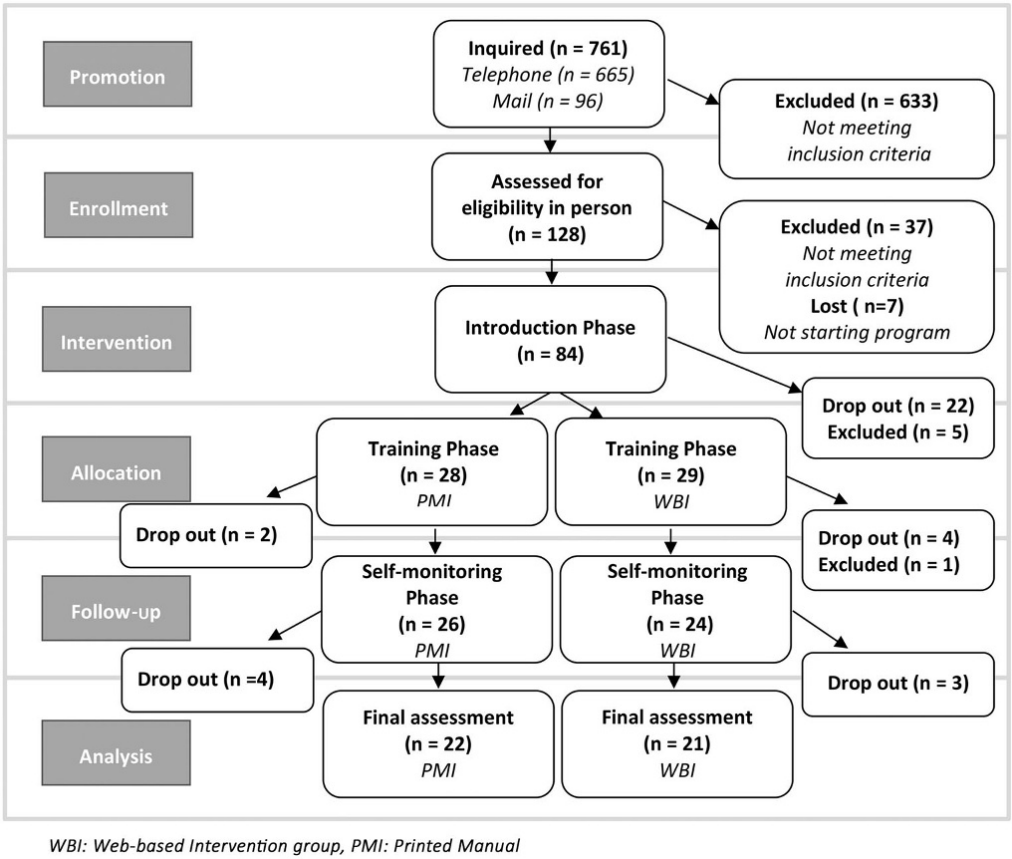
Study flow chart (*WBI* web-based intervention group, *PMI* printed manual group)

The study was conducted at the Women’s Health Center FEM Süd, located at the Kaiser Franz Josef Hospital in Vienna. Between September 2012 and September 2014, a total of 6 therapy groups enlisting 15 women each, were held in 3 consecutive waves. Most of the participants were recruited by newspaper announcements (52.4%) and via the internet (22.6%). Some were referred by family or friends (13.1%), 7.1% knew about the program by flyers and only 4.8% were informed by their practitioner. A total of 761 women inquired about participation by telephone or e‑mail: 633 were excluded for not meeting the inclusion criteria (Table 1) and received information about alternative treatment options and 128 women were assessed for eligibility in person and were informed about the study procedure. Based on this screening, another 37 applicants had to be referred to substitute programs and finally 91 candidates gave written consent to participate. With 7 women lost before the start of the intervention, data of 84 women could be assessed at the first group meeting. To create comparable conditions for evaluation of a follow-up intervention, all the participants had to complete the same treatment first. In this study, it required 4 months of group treatment and 2 months of training in using the aftercare delivery tool (either web-based or printed manual). The 57 participants could be allocated into 2 study arms, 6 subjects had to be excluded from starting with the aftercare intervention for no longer fulfilling inclusion criteria (e.g. inpatient treatment, quitting exercise sessions, long-term absence, missed measurements and pregnancy). There was an attrition of 28 participants before 6 months. After completion of the same pretreatment and the practical training 50 women started the follow-up phase and 7 participants dropped out during the self-monitoring phase. Final analyses for evaluation of the effects of the two different aftercare approaches could be calculated with data of 43 individuals completing the whole study (see study flow chart Fig. 1).

**Table 1:** Inclusion and exclusion criteria for study participation

Eligibility criteria	Inclusion criteria	Exclusion criteria
Sex	Women	Men
Age	18–80 years	<18 years
Body mass index (BMI)	Obesity grades I and II	Overweight, Obesity grade III
Participation	Attendance of all program modules	Selection of single program module
Informed consent	Written informed consent	Denial of written informed consent, denying permission to keep data after drop out
Attendance	70% attendance of face to face treatment, presence at assessment appointments	Long-term absence, missing assessment appointments
Physical requirements	Fit for moderate exercise	Severe physical impairments prohibiting even light exercise, pregnancy
Mental state/motivation	Mental stability, meeting group counselling requirements, motivation	Mental disorders with current symptoms, lack of motivation
Technical requirements	Internet access, basic computer skills	No Internet access, no computer experience
Validity	Study is the only current weight loss intervention	Simultaneous participation in additional weight reduction programs

### Intervention

This study was designed as a 2-armed randomized trial for obese women. Assessment was held at the beginning of the program, after 4 months (allocation), after 6 months (aftercare baseline) and after 1 year. Measured and self-reported data (questionnaires and exercise logs) were documented. During phase 1, the introduction phase, all participants attended an initial face to face life style intervention. Weekly group sessions were held by clinical psychologists or a nutritionist and concluded with 1h of instructed physical exercise. There were no additional one-to-one counselling sessions. With an allocation into two intervention groups the training phase (phase 2) started. All the participants still attended group sessions and physical exercise classes in a biweekly rhythm together, yet each intervention group was supplemented with a different aftercare delivery tool. The web-based intervention group (WBI) obtained a personal member login for the study webpage with biweekly gradual access to new information. The printed manual intervention group (PMI) received all the information and log templates as a printed tool kit at start of phase 2 and phase 3. The training phase was designed to support participants to get to know their aftercare intervention tool and reinforce autonomous self-monitoring. During the 6 months of self-monitoring (phase 3), there was no more face to face contact with the psychologist, individual interaction between participants and the program supervisor was enabled by telephone and e‑mail; however, the participants were encouraged to keep in touch with each other for further support. Electronic or printed inputs on nutrition and exercise needed for behavior change and modification of habits was structured in 12 biweekly modules. Coping strategies, such as mindfulness, body image and self-awareness were introduced to reduce stress-induced eating. Motivational book samples, including success stories in weight reduction were provided as vicarious experience. To elicit experimenting with more vegetables and fruits in a fat-reduced way of preparation, seasonal recipes were included in the aftercare intervention. Weekly exercise logs and documentation forms for weight and waist circumference should foster self-monitoring, further sustained by manuals for goal setting and progress evaluation charts. Monthly reminders were sent by the supervisor via e‑mail or regular mail. This update was structured into a personalized introduction, news with upcoming events, suggestions for objectives (e. g. healthy office lunch ideas) and concluded with an encouraging finish. Participants of the study were encouraged to contact the supervisor for tailored advice and progress feedback. This could be done by e‑mail, telephone or by data upload to the individual member accounts (accessible by the supervisor). After 12 months, a reunion was held at FEM Süd to increase attendance of the study participants at the last assessment. Incentives, such as a free presentation by an image consultant, a raffle to win a voucher and a healthy buffet were provided.

### Instruments

Exercise self-efficacy was evaluated with a questionnaire [17]. With 4 subscales the confidence in own ability to live an active life style (3 items), to start exercising (4 items), to keep up this form of active life style even in adverse conditions (11 items) and to restart exercise after a break (3 items) was assessed. An open question “what is most helpful to you, for maintaining or restarting exercise behavior?” supplemented this questionnaire. To measure and increase physical exercise, participants of the study used a pedometer.

Nutrition self-efficacy was measured with the Weight Efficacy Life-style questionnaire (WEL) [18]. The standardized questionnaire consists of 20 items with a 10-level Likert scale (0 = not confident, 9 = very confident) and 5 situational factors (negative emotions, availability, social pressure, physical discomfort and positive activities) assess the conviction in individual competence to resist eating in different situations. Translation from an English to a German version of the instrument was necessary and conducted according to acknowledged guidelines of translation [19].

### Measurements

Data were collected 4 times in each group. The first assessment was held at the first group meeting, followed by the data collection for allocation into intervention groups at 4 months. The baseline assessment for the follow-up intervention was held at 6 months and the final evaluation at 12 months. At each appointment anthropometric parameters (e.g. weight, height, waist circumference and percentage body fat) were measured and questionnaires as well as pedometer protocols (1 week) were collected.

### Allocation

At the end of the introduction phase, collected data were paired accordingly to the values of the domains in the WEL and the grade in the 4 subscales of the exercise self-efficacy questionnaire. Further markers regarded in this allocation were BMI, weight loss during the first 4 months and attendance of group sessions. Differences in sociodemographic characteristics of the final 43 participants were also reviewed and an even distribution was confirmed (see Table 2).

**Table 2:** Sociodemographic data of all participants starting the 6‑month follow-up intervention (including drop-outs between 6 and 12 months)

Characteristics	*p*-value	WBI (*n* = 21)		PMI (*n* = 22)	
*Age, years, mean (SD)*	*0.84*	*48.1*	*(±8.3)*	*48.8*	*(±13.0)*
*Level of education, n (%)*	*0.60*	*–*	*–*	*–*	*–*
Academic degree	–	5	23.8	6	27.3
High school degree	–	8	38.1	6	27.3
Grammar school	–	7	33.3	10	45.5
Compulsory school	–	1	4.8	0	0.0
*Status of employment, n (%)*	*0.67*	*–*	*–*	*–*	*–*
Full time job	–	12	57.1	11	50.0
Part time job	–	3	14.3	3	13.6
Retired	–	3	14.3	6	27.3
Continuing education	–	1	4.8	1	4.5
Maternity leave	–	1	4.8	0	0.0
Unemployed	–	1	4.8	0	0.0
Housewife	–	0	0.0	1	4.5
*Marital status, n (%)*	*0.52*	*–*	*–*	*–*	*–*
Single, no partner	–	4	19.0	4	18.2
In relationship	–	5	23.8	1	4.5
Married	–	8	38.1	10	45.5
Divorced	–	3	14.3	6	27.3
Widowed	–	1	4.8	1	4.5
*Children, n (%)*	*0.74*	*–*	*–*	*–*	*–*
No children	–	10	47.6	8	36.4
One child	–	6	28.6	7	31.8
Two children	–	5	23.8	7	31.8
*Single parent (with children <18 years), n (%)*	*0.58*	*1*	*4.8*	*2*	*9.1*
*Household income (net monthly), n (%)*	*0.56*	*–*	*–*	*–*	*–*
<€ 600	–	2	9.5	0	0.0
€ 601–900	–	0	0.0	1	4.5
€ 901–1200	–	1	4.8	2	9.1
€ 1201–1500	–	5	23.8	3	13.6
€ 1501–1800	–	1	4.8	4	18.2
€ 1801–2200	–	4	19.0	4	18.2
€ 2201–2600	–	4	19.0	3	13.6
>€ 2600	–	4	19.0	5	22.7
*BMI grade, n (%)*	*0.97*	*–*	*–*	*–*	*–*
Overweight	–	3	14.3	3	13.6
Obesity grade I	–	9	42.9	11	50.0
Obesity grade II	–	7	33.3	6	27.3
Obesity grade III	–	2	9.5	2	9.1

*WBI* web-based intervention group, *PMI* printed manual intervention group, *BMI* body mass index

### Statistical analysis

All statistical analyses were performed using the SPSS for Windows software, version 19.0. In this study, data were collected 4 times in each intervention group; however, there were only 2 measurements before and after the follow-up intervention. Mixed model ANOVAs were calculated for completers with available data at 6 and 12 months. In addition, calculations using engagement in the follow-up support as a further factor comparing the two interventions were performed. For all tests performed *p*‑values of *p* <0.05 were regarded as statistically significant.

## Results

**Fig. 2:**
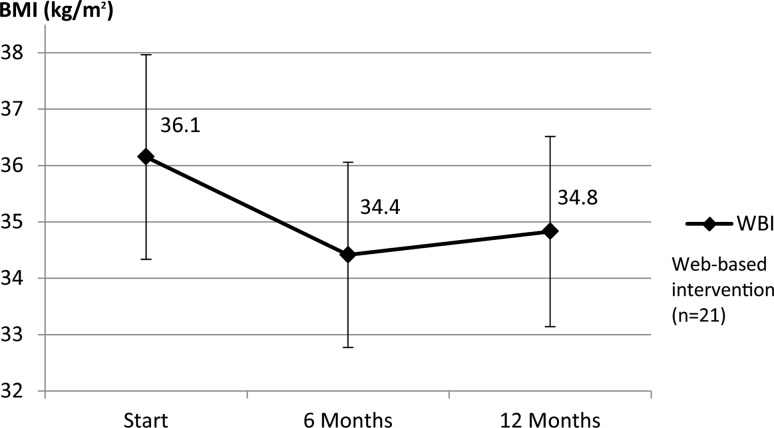
Mean (±SD) body mass index (BMI) at start, after the introduction and training phase (6 months) and after follow-up (12 months) using a web-based intervention

**Fig. 3:**
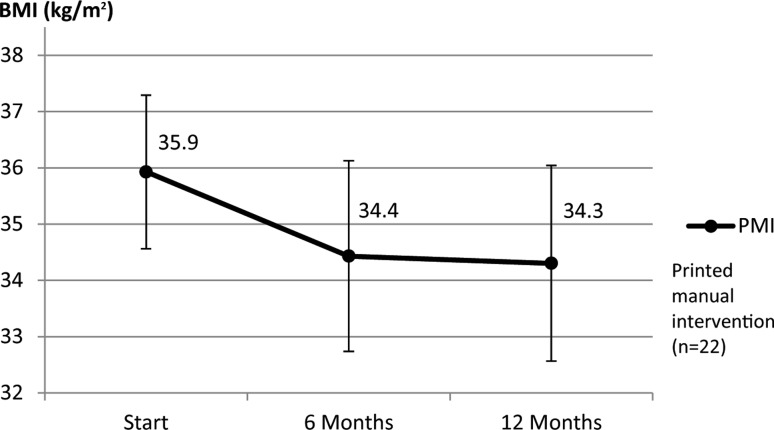
Mean (±SD) body mass index (BMI) at start, after the introduction and training phase (6 months) and after follow-up (12 months) using a printed manual intervention

In this study, after a significant reduction of anthropometric data during the face to face treatment, values could be maintained within the predefined range until the final evaluation (Figs. 2 and 3) in each intervention group. There were no significant differences in progress between the WBI or the PMI groups (BMI/month: WBI = +0.07 kg/m^2^, PMI = −0.02 kg/m^2^, *p* = 0.258; body fat/month: WBI = +0.05%, PMI = +0.03%, *p* = 0.916). Both study arms profited from training, with no significant differences by the method (WBI or PMI). The initial 6 months produced a significant increase in self-efficacy values in both groups. Subscales of exercise self-efficacy active lifestyle (mean WBI = +0.14, mean PMI = +0.10, *p* = 0.032), keep up exercising (mean WBI = +0.23, mean PMI = +0.21, *p* ≤ 0.001) and resume exercising (mean WBI = +0.44, mean PMI = +0.30, *p* ≤ 0.001) all improved. There was a significant increase in scores of nutrition self-efficacy in program completers during the first 6 months (average increase WBI = +2.08 points, average increase PMI = +1.37 points, *p* ≤ 0.001). At the 12-month assessment, values of nutrition self-efficacy had stabilized close to the values before the self-monitoring phase. On the other hand, a slight decrease in exercise self-efficacy could be documented after termination of group sessions with mandatory attendance of exercise classes; however, this decline was significant only for the subscale resume exercising (mean WBI = −0.25, mean PMI = −0.20, *p* = 0.048) with no superiority of any intervention group (active lifestyle mean WBI = −0.09, mean PMI = −0.10, *p* = 0.219, start exercising mean WBI = −0.11, mean PMI = ±0.0, *p* = 0.630 and keep up exercising mean WBI = −0.02, mean PMI = −0.11, *p* = 0.369). Further analysis surveying specifics of the study arms explored possible variations in utilization and acceptance of the provided method (WBI/PMI). No significant differences between both intervention groups could be found when engagement in the follow-up support was calculated as a further factor (Tables 3 and 4). Feedback on the WBI showed a slightly higher percentage in acceptability: 81.0% of participants in the WBI stated to have been content with the allocation to their intervention group, whereas only 55.0% felt the same in the PMI (*p* = 0.074). Furthermore, participants of the WBI reported a more frequent contact with the program supervisor (always/often: WBI = 20%, PMI = 4.8%, sometimes: WBI = 45%, PMI = 23.8%, *p* = 0.054).

**Table 3:** Mixed model ANOVA for nutrition self-efficacy (6–12 months) by intervention group (web-based/printed manual) and engagement in follow-up support

Domain	Time	Group	Usage	Mean^a^	SD	Group	Usage	Mean^b^	SD	Main effects			2-way interaction			3-way interaction
										*P* Time	*P* Group	*P* Usage	*P* Time * Group	*P* Time * Usage	*P* Group * Usage	*P* Time * Group * Usage
Self-efficacy negative emotions	6 months	WBI	Yes (*n* = 14)	7.46	1.26	WBI	Yes (*n* = 14)	0.00	1.49	0.814	0.958	0.614	0.381	0.532	0.597	0.208
			No (*n* = 7)	6.82	1.37											
		PMI	Yes (*n* = 13)	7.10	1.57		No (*n* = 7)	0.29	1.58							
			No (*n* = 9)	7.53	1.72											
	12 months	WBI	Yes (*n* = 14)	7.46	1.86	PMI	Yes (*n* = 13)	0.17	1.39							
			No (*n* = 7)	7.11	1.66											
		PMI	Yes (*n* = 13)	7.27	1.80		No (*n* = 9)	−0.67	1.00							
			No (*n* = 9)	6.86	1.95											
Self-efficacy availability	6 months	WBI	Yes (*n* = 14)	6.80	2.13	WBI	Yes (*n* = 14)	0.02	1.63	0.237	0.837	0.095	0.620	0.139	0.629	0.786
			No (*n* = 7)	6.54	1.06											
		PMI	Yes (*n* = 13)	6.90	1.69		No (*n* = 7)	−0.75	0.99							
			No (*n* = 9)	6.00	1.64											
	12 months	WBI	Yes (*n* = 14)	6.82	2.02	PMI	Yes (*n* = 13)	0.12	1.18							
			No (*n* = 7)	5.79	1.56											
		PMI	Yes (*n* = 13)	7.02	1.67		No (*n* = 9)	−0.42	1.36							
			No (*n* = 9)	5.58	2.02											
Self-efficacy social pressure	6 months	WBI	Yes (*n* = 14)	6.93	2.24	WBI	Yes (*n* = 14)	0.50	2.00	0.915	0.821	0.495	0.682	0.105	0.618	0.438
			No (*n* = 7)	7.39	1.30											
		PMI	Yes (*n* = 13)	7.17	1.75		No (*n* = 7)	−0.64	1.43							
			No (*n* = 9)	6.69	2.03											
	12 months	WBI	Yes (*n* = 14)	7.43	1.98	PMI	Yes (*n* = 13)	0.33	0.83							
			No (*n* = 7)	6.75	2.24											
		PMI	Yes (*n* = 13)	7.50	1.63		No (*n* = 9)	−0.08	1.22							
			No (*n* = 9)	6.61	2.22											
Self-efficacy physical discomfort	6 months	WBI	Yes (*n* = 14)	7.59	1.07	WBI	Yes (*n* = 14)	0.14	1.07	0.940	0.896	0.586	0.296	0.231	0.292	0.504
			No (*n* = 7)	7.71	0.68											
		PMI	Yes (*n* = 13)	6.98	1.59		No (*n* = 7)	−0.57	1.47							
			No (*n* = 9)	7.81	1.48											
	12 months	WBI	Yes (*n* = 14)	7.73	1.56	PMI	Yes (*n* = 13)	0.29	1.42							
			No (*n* = 7)	7.14	1.94											
		PMI	Yes (*n* = 13)	7.27	1.95		No (*n* = 9)	0.08	0.63							
			No (*n* = 9)	7.89	1.45											
Self-efficacy positive activities	6 months	WBI	Yes (*n* = 14)	7.27	1.67	WBI	Yes (*n* = 14)	0.41	1.19	0.698	0.572	0.783	0.402	0.141	0.950	0.710
			No (*n* = 7)	7.68	0.77											
		PMI	Yes (*n* = 13)	7.67	1.33		No (*n* = 7)	−0.25	0.60							
			No (*n* = 9)	8.00	0.90											
	12 months	WBI	Yes (*n* = 14)	7.68	1.43	PMI	Yes (*n* = 13)	−0.02	1.15							
			No (*n* = 7)	7.43	1.12											
		PMI	Yes (*n* = 13)	7.65	1.19		No (*n* = 9)	−0.42	1.21							
			No (*n* = 9)	7.58	1.39											
General nutrition self-efficacy	6 months	WBI	Yes (*n* = 14)	7.21	1.40	WBI	Yes (*n* = 14)	0.21	1.17	0.638	0.958	0.565	0.938	0.091	0.920	0.844
			No (*n* = 7)	7.23	0.85											
		PMI	Yes (*n* = 13)	7.17	1.26		No (*n* = 7)	−0.39	0.97							
			No (*n* = 9)	7.21	1.43											
	12 months	WBI	Yes (*n* = 14)	7.42	1.56	PMI	Yes (*n* = 13)	0.17	0.85							
			No (*n* = 7)	6.84	1.47											
		PMI	Yes (*n* = 13)	7.34	1.35		No (*n* = 9)	−0.30	0.81							
			No (*n* = 9)	6.91	1.57											

*WBI/PMI* Intervention group (web-based/printed manual), *Usage* engagement in follow-up support, *WBI* web-based intervention, *PMI* printed manual intervention

*Mean* ^a^ mean item score per domain

*Mean* ^b^ increase/decrease of mean item score (6–12 months)

**Table 4:** Mixed model ANOVA for exercise self-efficacy (6–12 months) by intervention group (web-based/printed manual) and engagement in follow-up support

Domain	Time	Group	Usage	Mean^a^	SD	Group	Usage	Mean^b^	SD	Main effects			2-way interaction			3-way interaction
										*P* Time	*P* Group	*P* Usage	*P* Time * Group	*P* Time * Usage	*P* Group * Usage	*P* Time * Group * Usage
Self-efficacy active lifestyle	6 months	WBI	Yes (*n* = 14)	3.50	0.43	WBI	Yes (*n* = 14)	0.12	0.41	0.042	0.858	0.486	0.616	0.010	0.115	0.158
			No (*n* = 7)	3.48	0.33											
		PMI	Yes (*n* = 13)	3.36	0.46		No (*n* = 7)	−0.53	0.74							
			No (*n* = 9)	3.59	0.43											
	12 months	WBI	Yes (*n* = 14)	3.62	0.37	PMI	Yes (*n* = 13)	−0.03	0.48							
			No (*n* = 7)	2.95	0.71											
		PMI	Yes (*n* = 13)	3.33	0.69		No (*n* = 9)	−0.22	0.37							
			No (*n* = 9)	3.37	0.73											
Self-efficacy start exercising	6 months	WBI	Yes (*n* = 14)	3.45	0.67	WBI	Yes (*n* = 14)	−0.24	0.68	0.862	0.294	0.764	0.834	0.368	0.016	0.483
			No (*n* = 7)	2.79	0.91											
		PMI	Yes (*n* = 13)	3.10	0.53		No (*n* = 7)	0.14	1.33							
			No (*n* = 9)	3.44	0.62											
	12 months	WBI	Yes (*n* = 14)	3.21	0.66	PMI	Yes (*n* = 13)	−0.02	0.54							
			No (*n* = 7)	2.93	0.61											
		PMI	Yes (*n* = 13)	3.08	0.66		No (*n* = 9)	0.03	0.29							
			No (*n* = 9)	3.47	0.48											
Self-efficacy keep up exercising	6 months	WBI	Yes (*n* = 14)	3.12	0.69	WBI	Yes (*n* = 14)	−0.02	0.54	0.325	0.891	0.588	0.519	0.503	0.414	0.617
			No (*n* = 7)	3.08	0.21											
		PMI	Yes (*n* = 13)	3.00	0.43		No (*n* = 7)	−0.04	0.53							
			No (*n* = 9)	3.34	0.70											
	12 months	WBI	Yes (*n* = 14)	3.10	0.74	PMI	Yes (*n* = 13)	−0.03	0.49							
			No (*n* = 7)	3.04	0.55											
		PMI	Yes (*n* = 13)	2.97	0.65		No (*n* = 9)	−0.21	0.21							
			No (*n* = 9)	3.13	0.77											
Self-efficacy resume exercising	6 months	WBI	Yes (*n* = 14)	3.60	0.59	WBI	Yes (*n* = 14)	−0.34	0.68	0.103	0.924	0.592	0.857	0.279	0.265	0.944
			No (*n* = 7)	3.38	0.52											
		PMI	Yes (*n* = 13)	3.38	0.45		No (*n* = 7)	−0.09	0.81							
			No (*n* = 9)	3.52	0.71											
	12 months	WBI	Yes (*n* = 14)	3.26	0.63	PMI	Yes (*n* = 13)	−0.30	0.93							
			No (*n* = 7)	3.29	0.52											
		PMI	Yes (*n* = 13)	3.08	0.77		No (*n* = 9)	−0.04	0.26							
			No (*n* = 9)	3.48	0.71											

*WBI/PMI* intervention group (web-based/printed manual), *Usage* engagement in follow-up support, *WBI* web-based intervention, *PMI* printed manual intervention

*Mean* ^a^ mean item score per domain

*Mean* ^b^ increase/decrease of mean item score (6–12 months)

## Discussion

In this study, there were no differences found between the groups in their change of exercise self-efficacy. The significant increase in values during the face to face treatment was followed by an expected slight decrease after termination of group sessions with mandatory attendance of exercise classes in both groups. Similar WEL values for both intervention groups showed a significant increase in nutrition self-efficacy during the first 6 months, followed by no further significant improvements or impairments at 12 months. Measured anthropometric data also did not differ in the reduction during the face to face treatment and a slow but not significant subsequent increase over both groups at 12 months. On the other hand, self-assessment indicated a slight preference towards the WBI with higher scores in acceptability.

Studies have repeatedly emphasized the value of social support in initiating and even more in maintaining weight loss [4]. It is even a well-known predictor for success. Stabilizing weight can be especially demanding due to diminishing positive external feedback [11, 20]. In the present evaluation, none of the intervention groups showed a decrease in their values of the subscale social pressure (mean WBI = +0.1, mean PMI = +0.2, *p* = 0.546) during the self-monitoring phase. The importance of allies in the therapy group and the ongoing support by the program supervisor was repeatedly mentioned by study participants. A sense of solidarity experienced in the group sessions might even have been preserved to a degree with the aftercare intervention. Especially participants of the WBI reported to have used the opportunity of keeping in touch with the program supervisor more frequently. Regarding the importance of social support in weight stabilization, technical solutions to foster communication and the feeling of belonging, seem to be beneficial.

Answers to open questions in the program evaluation and the slight decrease in the WEL subscale availability (mean WBI = −0.2, mean PMI = −0.1, *p* = 0.418) in both intervention groups also indicated a tendency to rely on external factors. Study participants relied more on reminders than autonomous work with the provided progress forms. Repeated revisions of exercise instructions were preferred over independent recording or memorizing sequences of the attended exercise classes. Technical solutions make these continuing cues for self-monitoring in a long-term follow-up support feasible [16, 20, 21]. On another level, this inclination towards an external locus of control is in accordance with another common impediment in weight loss. Low self-efficacy to resist temptation when food is available is especially challenging in today’s obesogenic environment. Studies of workplace health promotion or of school catering document the excess of unhealthy food available and the lack of healthy alternatives as one of the barriers in daily nutrition choices [22, 23]. Especially women in low-wage jobs have to face additional barriers, such as the lack of time or possibility to retreat for breaks, a topic which was repeatedly discussed during group sessions. All these results emphasize the importance of developing public health strategies to change the obesogenic environment to support individual behavior change [23–26].

Calculations of the impact of engagement in the follow-up support tools with mixed model ANOVAs did not find significant differences between the subgroups. Nutrition self-efficacy scores (Table 3) of users (WBI *n* = 14, PMI *n* = 13) were higher than of non-users (WBI *n* = 7, PMI *n* = 9) of either aftercare intervention but maybe due to the small sample size, this trend was not significant. Additional qualitative analysis could be helpful to interpret results, such as the one exception in the subscale negative emotions. Participants who had not used the WBI (*n* = 7) showed a better progress in scores than women who had use the aftercare support (*n* = 14). The higher but not significant loss of users (WBI *n* = 14, PMI *n* = 13) in scores of exercise self-efficacy subscales (start exercising and resume exercising) in both intervention groups might be explained by the awareness of barriers to action (Table 4). Working with exercise logs might have increased introspection. This interpretation is in line with other studies, establishing the need for self-monitoring to prevent overestimating own physical activity [27].

One advantage of this evaluation was the implementation of the study in a women’s health center. The constant demand for long-term, low cost obesity treatment (including nutrition, exercise and psychological support in one intervention) led to a bottom-up approach. With the evaluation of a web-based follow-up support observing scientific standards, the request for follow-up support and technological improvements had been acknowledged. Especially for working mothers and women with multiple demands, the evaluation of a time-saving, location independent delivery of aftercare was promising. The gender-specific approach of the intervention was a perfect fit with the setting in a women-only environment. Body insecurity and cultural demands in exercise classes were no issue with the obese study population. The option of affordable exercise classes in a familiar environment, was an additional bonus some participants took advantage of with much enthusiasm, even 2 years after the end of the study.

A major limitation of this study was the lack of a waiting list control group. The small sample size available for evaluation of the follow-up support proved problematic with some subgroup analyses and together with the inclusion criteria the generalizability of results is restricted. Longer follow-up support and further measurements might have given better insights into treatment outcomes. Rapid technical improvements with modernized self-monitoring tools and smartphones could not be incorporated into the study. This compromised convenience of the follow-up support and the social interaction within the group.

## Conclusion

This study with two different forms of aftercare delivery, following an initial face to face life style treatment, did not produce significant differences between the WBI and the PMI. There was no superiority in values of nutrition and exercise self-efficacy in any one of the intervention groups. After a significant decrease in anthropometric parameters and a significant increase in self-efficacy values, neither WBI nor PMI produced further significant changes during the last 6 months of the trial. Nevertheless, the web-based support seemed to facilitate contact with the program supervisor and received high acceptability ratings, which is a valuable effect in long-term treatment. No significant differences regarding the engagement in the follow-up tools could be found, although subgroup analysis indicated the positive effect of involvement in either form of aftercare support. With the need for long-term intervention strategies in obesity treatment, these results encourage efforts to pursue the development of technological add-on concepts to standard care. Incorporated electronic health concepts have the potential to make long-term support more convenient and increase appeal.
